# Trends and Disparities in Dementia Among Older Adults With Coronary Artery Disease, 1999–2023: Insights From the CDC WONDER Database

**DOI:** 10.1002/brb3.71125

**Published:** 2025-12-17

**Authors:** Saifullah Khan, Muhammad Hassan, Muhammad Hussain, Wania Fatima, Javeria Nawaz, F. N. U. Pirih, Aiza Ahsan, Nisha Khalid, Mariam Qaisar, Sherif Eltawansy, Muhammad Khalid Afridi, Preet Memon, Raheel Ahmed, Hasibullah Aminpoor

**Affiliations:** ^1^ Department of Medicine Dow University of Health Sciences Karachi Pakistan; ^2^ Department of Medicine People's University of Medical and Health Sciences Nawabshah Pakistan; ^3^ Department of Medicine Jersey Shore University Medical Center Neptune New Jersey USA; ^4^ Department of Cardiology, Newcastle Upon Tyne Hospitals NHS Foundation Trust Newcastle Upon Tyne UK; ^5^ Faculty of Medicine Kabul University of Medical Sciences “Abu Ali Ibn Sina ” Kabul Afghanistan

## Abstract

**Introduction:**

Despite growing multimorbidity rates, older adults, particularly those with dementia, remain underrepresented in cardiovascular research. Most research on CAD excludes individuals with cognitive impairment, leaving a gap in our understanding of how dementia and CAD affect mortality. This study bridges that gap by examining 25‐year nationwide trends in dementia and CAD‐related mortality among elderly in the United States.

**Methods:**

We performed a descriptive trend analysis using Centers for Disease Control and Prevention's Wide‐Ranging Online Data for Epidemiologic Research (CDC WONDER) Multiple Cause‐of‐Death data (1999–2023). Deaths with International Classification of Diseases, 10th Revision (ICD‐10) codes I20–I25 (CAD) and F01, F03, G30 (dementia) were included if either appeared anywhere on the death certificate. Age‐adjusted mortality rates (AAMRs) per 100,000 population were calculated and stratified by sex, race/ethnicity, state, census region, place of death, and urban/rural status. Average AAMRs were calculated by using the AVERAGE Excel function for the entire study period. Joinpoint regression with annual percentage change (APC), average APC (AAPC), and 95% confidence intervals (CIs) were used to evaluate temporal changes. The threshold for statistical significance was set at *p* < 0.05.

**Results:**

In adults aged ≥65 years, dementia with CAD was the cause of 1,256,284 deaths between 1999 and 2023. More than half took place in long‐term care institutions. Overall, the AAMR trend showed no statistically significant change (AAPC: −0.23; *p* = 0.325). The average AAMRs of males were greater than those of females (127.75 vs. 109.83). Non‐Hispanic (NH) Whites had the highest average AAMRs (121.59). Regionally, average AAMRs were highest in the Midwest (123.02), followed by Northeast, South, and West. Average AAMRs were highest in rural areas (129.73 vs. 117.12 in urban areas) with a statistically significant upward trend in rural AAMRs (AAPC: 1.16; *p* < 0.001), whereas urban rates remained stable. Subgroup analysis revealed highest mortality burden associated with vascular dementia‐related CAD (AAPC = 8.49).

**Conclusion:**

Among the elderly, dementia with CAD continues to cause significant mortality in the United States, particularly in males, rural regions, and long‐term care settings. These findings highlight an urgent need for inclusive cardiovascular research and health care personalized to this often‐overlooked population.

## Graphical abstract

From 1999 to 2023, over 1.25 million US deaths involved both dementia and coronary artery disease. Mortality was highest among males, rural residents, and long‐term care patients, with rising rural trends. The findings call for inclusive cardiovascular research and tailored care for elderly individuals with cognitive impairment.

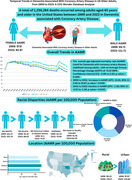

## 1 Introduction

World Health Organization (WHO) defines dementia as a condition that can arise from various diseases that progressively harm nerve cells and impair brain function (WHO 2025). This usually results in a decline in cognitive abilities, which is greater than what is typically expected from normal aging processes (WHO 2025). Alzheimer's disease is the leading form of dementia, responsible for 60%–70% of all cases (WHO 2025). CAD is characterized by the development of atherosclerosis in the coronary arteries, and it can sometimes occur without any noticeable symptoms (Shahjehan et al. 2025).

In 2022, dementia was the underlying cause of death for 288,436 adults aged 65 and older in the United States (Kramarow and Betzaida 2024). CAD is the most common heart condition in the United States, remaining a leading cause of death. According to the American Heart Association (AHA), as of 2018, approximately 16.5 million adults aged 20 and older in the United States were living with CAD (Regmi and Siccardi 2025).

Research indicates that poor cardiovascular health in older adults is a significant predictor of transitions to cognitive impairment, dementia, and increased mortality rates (Georgescu et al. 2025). For instance, a study involving a Danish cohort of patients aged 65 and older identified CAD as a modest risk factor for the onset of dementia (Olesen et al. 2025). Similarly, findings from the Cardiovascular Health Study revealed that older adults with existing CAD tend to experience a higher incidence of dementia (Newman et al. 2005). Furthermore, a meta‐analysis of longitudinal studies encompassing approximately 1.3 million individuals demonstrated that a history of coronary heart disease (CHD) is associated with a 27% increased risk of developing dementia (Wolters et al. 2018).

Despite increased awareness of the link between CAD and dementia, few studies have investigated mortality trends in older adults with CAD‐related dementia. Understanding these trends is vital, especially given the aging population and changing patterns of chronic diseases. This study seeks to address this gap by analyzing mortality patterns in older adults diagnosed with CAD‐related dementia, offering valuable insights for clinicians and public health policymakers.

## 2 Methods

### 2.1 Study Design and Data Source

This study utilizes mortality data from the Centers for Disease Control and Prevention's Wide‐Ranging Online Data for Epidemiologic Research (CDC WONDER) database (Friede et al. 1993), covering the period from 1999 to 2023. Information on deaths was obtained from US death certificates compiled in the Multiple Cause‐of‐Death public‐use dataset, which includes records from all 50 states and the District of Columbia. The CDC WONDER system is a well‐established resource in epidemiological investigations for analyzing nationwide mortality patterns.

### 2.2 Study Population

Mortality data were determined using the International Classification of Diseases, 10th Revision (ICD‐10) codes: I20–I25 for coronary artery disease (Saad et al. 2025; Sohail et al. 2025) and F01, F03, and G30 for dementia (Ali et al. 2024; Sohail et al. 2025). Deaths were considered eligible if the specified condition was recorded anywhere on the death certificate, either as the underlying cause or as a contributing factor.

### 2.3 Data Abstraction

The variables extracted included year of death; demographic factors, such as age, sex, and race/ethnicity; place and geographic region of death; US state; and urban–rural classification. A subgroup analysis was conducted by dementia subtype to evaluate mortality trends for vascular dementia (F01), Alzheimer's disease (G30), and unspecified dementia (F03) in association with coronary artery disease (I20–I25). Each subtype was analyzed separately using identical Joinpoint regression procedures. Age was analyzed in 10‐year intervals for individuals aged 65 years and older. Race and ethnicity data were harmonized across 1999–2023 for consistency. From 2018 onward, CDC WONDER introduced a six‐category system; therefore, “NH Native Hawaiian or Other Pacific Islander” and “NH Asian” were merged into a single NH Asian or Pacific Islander’ group to match the earlier classification. Race/Ethnicity categories comprised NH White, NH Black or African American, Hispanic or Latino, NH American Indian or Alaska Native, and NH Asian or Pacific Islander. Place of death was classified as occurring in medical facilities (including outpatient settings, emergency departments, inpatient wards, or cases recorded as dead on arrival or of unknown status), at home, in hospice, or in long‐term care facilities.

Geographic regions followed US Census Bureau classifications (Northeast, Midwest, South, and West). Urban–rural status was assigned using the 2013 National Center for Health Statistics Urban–Rural Classification Scheme (Ingram and Franco 2014). This study was conducted and reported in accordance with the STROBE (Strengthening the Reporting of Observational Studies in Epidemiology) guidelines (von Elm et al. 2007), and a completed STROBE checklist is provided as Table S11.

### 2.4 Statistical Analysis

Age‐adjusted mortality rates (AAMRs) per 100,000 population were calculated annually and stratified by sex, race/ethnicity, US state, and urban–rural classification, with 95% confidence interval (CI). Age adjustment was performed using the direct standardization method, referencing the 2000 US standard population. For each demographic subgroup, the average AAMR (1999–2023) was calculated as the arithmetic mean of annual AAMRs across the 25‐year period to provide a summary measure of mortality level. Temporal trends in AAMRs were analyzed with the Joinpoint Regression Program (version 5.4.0; National Cancer Institute) (Joinpoint Regression Program 2025), which fits log‐linear models to detect statistically significant inflection points in trends. Model selection was performed using the weighted Bayesian information criterion (BIC), and 95% CIs for the annual percent change (APC) and average APC (AAPC) were estimated using the empirical quantile method. APC estimates and corresponding 95% CIs were obtained from the model. Statistical significance was defined as a two‐sided *p* value < 0.05.

## 3 Results

Between 1999 and 2023, dementia with CAD resulted in a total of 1,256,284 fatalities among older adults aged 65 years and more in the United States (Table S1). This death toll was prevalent across various places, with the leading most occurring in nursing homes (53.4%), 19.5% in medical facilities, 18.1% in the decedent's homes, 5.2% at other locations, and 3.7% in hospice facilities (Table S2).

### 3.1 Annual Trends for Dementia With Coronary Artery Disease‐Related AAMR Among Older Adults

**FIGURE 1:**
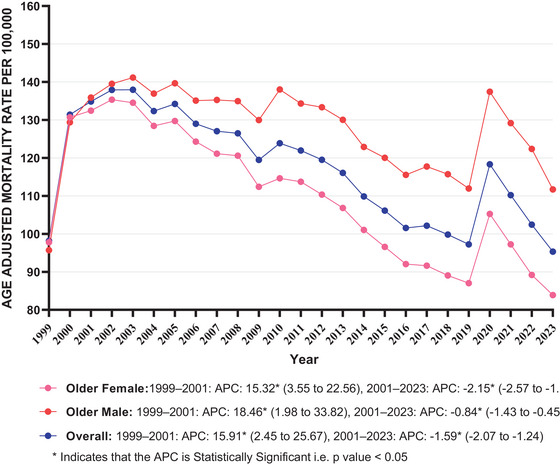
Dementia with coronary artery disease in older adults AAMR stratified by overall and sex per 100,000 population. APC, annual percentage change.

The overall AAMR for dementia with coronary artery disease–related deaths among adults decreased from 98.18 (95% CI: 97.13–99.23 in 1999) to 95.32 (95% CI: 94.47–96.16 in 2023) (Table S10). However, the overall trend in AAMR showed no statistically significant change between 1999 and 2023 with an AAPC of −0.23 (95% CI: −0.99 to 0.40; *p* value = 0.325). AAMR trend exhibited a rise between 1999 and 2001, followed by a decrease in trend from 2001 to 2023 (Table S3; Figure 1).

### 3.2 Dementia With Coronary Artery Disease–Related AAMR Stratified by Sex Among Older Adults

Older males exhibited slightly higher average AAMRs compared to older females (average AAMR for older men: 127.75, 95% CI: 125.96–129.54; for older women: 109.83, 95% CI: 108.58–111.09). The AAMR trend remained stable for both sexes; however, among males, there was no statistically significant change from 1999 to 2023 (older males: AAPC: 0.63 [95% CI: −0.23 to 1.63; *p* value = 0.108]; older females: AAPC: −0.80, [95% CI: −1.46 to −0.32; *p* value = 0.001]).

The AAMR trend for older males increased from 1999 to 2001, followed by a period of stabilization in AAMR trend between 2001 and 2023. Among older females, trend in AAMR manifested an increase between 1999 and 2001, after which AAMR showed a decline from 2001 to 2023 (Table S4; Figure 1).

### 3.3 Dementia With Coronary Artery Disease–Related AAMR Stratified by Race/Ethnicity Among Older Adults

The highest average AAMRs were recorded among NH Whites, with considerable differences among the remaining racial/ethnic groups which included NH Black, Hispanic, NH American Indian or Alaskan Native, and NH Asian or Pacific Islander (average AAMR: NH White: 121.59 [95% CI: 120.44–122.75]; NH Black: 116.32 [95% CI: 112.60–120.04]; Hispanic: 89.82 [95% CI: 86.00–93.64]; NH American Indian or Alaskan Native: 84.88 [95% CI: 70.83–99.36]; NH Asian or Pacific Islander: 57.31 [95% CI: 53.08–61.54]).

The AAMR trend of all races remained stable between 1999 and 2023, showing no statistically significant differences observed (*p* > 0.05) (NH White: AAPC: −0.10 [95% CI: −0.75 to 0.47; *p* value = 0.593]; NH Black AAPC: −0.20 [95% CI: −1.45 to 0.85; *p* value = 0.539]; Hispanic: AAPC: 0.19 [95% CI: −0.57 to 1.13; *p* value = 0.628]; NH American Indian or Alaskan Native: AAPC: 0.23 [95% CI: −0.59 to 1.90; *p* value = 0.505]; NH Asian or Pacific Islander: AAPC: 0.18 [95% CI: −2.06 to 2.53; *p* value = 0.893]).

**FIGURE 2:**
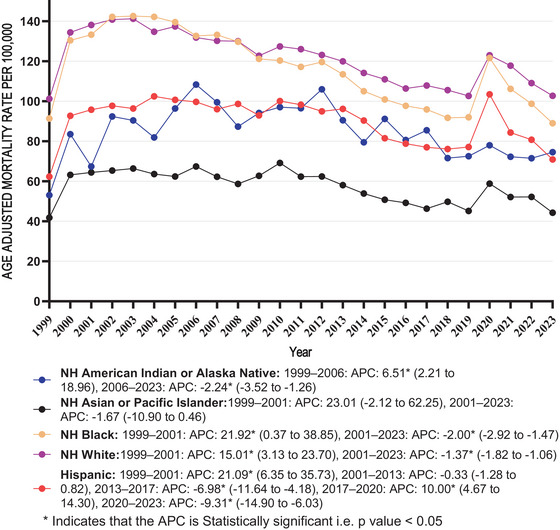
Dementia with coronary artery disease in older adults AAMR stratified by race per 100,000 population. APC, annual percentage change.

From 1999 to 2001, NH White individuals demonstrated a rise in AAMR trend, followed by a decline by 2023. In NH Black individuals, AAMR trend increased from 1999 to 2001, followed by a decline from 2001 to 2023. Among Hispanic individuals, there was a rise from 1999 to 2001, followed by a non‐significant change from 2001 to 2013. Between 2013 and 2017, a decline was observed, which then proceeded to an increase from 2017 to 2020. By 2023, AAMR trend increased. Among NH American Indian or Alaska Native individuals, there was an increase from 1999 to 2006, followed by a drop from 2006 to 2023. In NH Asian or Pacific Islander individuals, an increase was observed between 1999 and 2001, followed by a downward trend from 1999 to 2023 (Table S5; Figure 2).

### 3.4 Dementia With Coronary Artery Disease–Related AAMR Stratified by Geographical Regions Among Older Adults

#### 3.4.1 Stratified by Census

During the study duration, the highest average AAMRs were observed in the Midwest (AAMR: 123.02; 95% CI: 120.83–125.21), followed by the Northeast (average AAMR: 117.44; 95% CI: 115.18–119.70), South (average AAMR: 117.28; 95% CI: 115.54–119.02), and West regions (average AAMR: 110.48; 95% CI: 108.28–112.67).

The AAMR of all regions manifested a trend of stabilization with no statistically significant change (*p* value > 0.05) between 1999 and 2023, except the West, which showed a statistically significant trend (*p* value < 0.05) (Midwest: AAPC = −0.10 [95% CI: −0.91 to 0.59; *p* value = 0.612]; South: AAPC = −0.14 [95% CI: −0.56 to 0.28; *p* value = 0.394]; Northeast: AAPC = −0.19 [95% CI: −1.10 to 0.44; *p* value = 0.381]; West: AAPC = −0.58 [95% CI: −1.13 to −0.09; *p* value = 0.05]).

**FIGURE 3:**
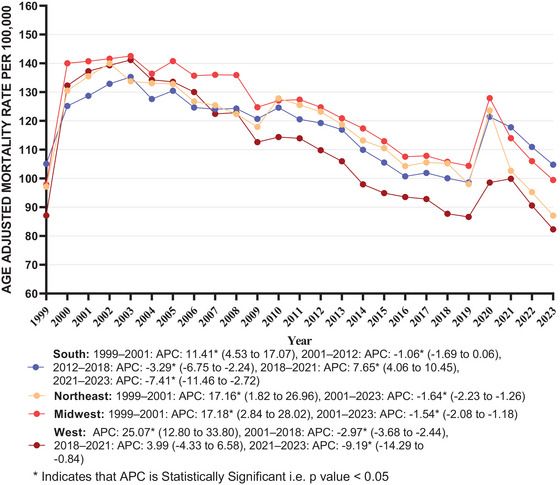
Dementia with coronary artery disease in older adults AAMR stratified by US census per 100,000 population. APC, annual percentage change.

In the Midwest, AAMR rose from 1999 to 2001, followed by a decline from 2001 to 2023. In the South, there was an elevation in AAMR trend from 1999 to 2001, followed by a decline from 2001 to 2012. From 2012 to 2018, a decline was observed, which increased by 2021 and declined from 2021 to 2023. From 1999 to 2001, the Northeast showed a rise, followed by a drop from 2001 to 2023. In the West, there was an upward trend from 1999 to 2001, followed by a decrement from 2001 to 2018. AAMR trend showed a non‐significant increase from 2018 to 2021, followed by a downward trend from 2021 to 2023 (Table S6; Figure 3).

#### 3.4.2 Stratified by Urbanization Region

Rural areas showed slightly higher average AAMRs throughout the study period, with an average AAMR of 129.73 for rural (95% CI: 127.15–132.31) and 117.12 for urban (95% CI: 115.96–118.27). The AAMR trend of rural areas increased from 1999 to 2020 (Rural: AAPC = 1.16 [95% CI: 0.72–1.59; *p* value < 0.001]). The AAMR in urban areas demonstrated a non‐significant plateaued trend between 1999 and 2020 (Urban: AAPC = 0.30 [95% CI: −0.32 to 0.92; *p* value = 0.347]).

**FIGURE 4:**
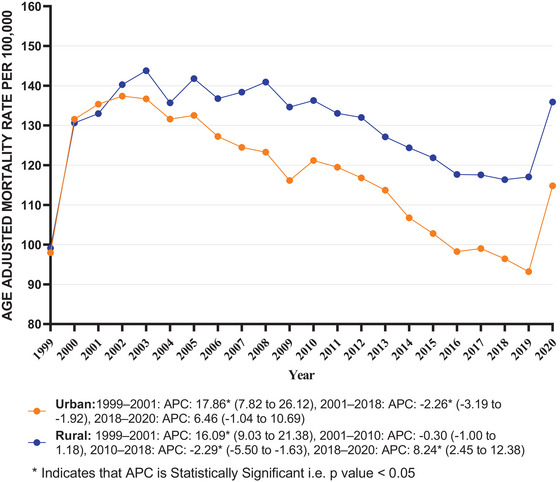
Dementia with coronary artery disease in older adults AAMR stratified by urbanization per 100,000 population. APC, annual percentage change.

In urban areas, there was a dramatic rise from 1999 to 2001, followed by a decrease from 2001 to 2018. From 2018 to 2020, AAMR trend showed a rise. In rural areas, there was an upward trend from 1999 to 2001, followed by a non‐significant change from 2001 to 2010. From 2010 to 2018, a significant decline was observed, which then shifted to an increase from 2018 to 2020 (Table S7; Figure 4).

#### 3.4.3 Stratified by State

**FIGURE 5:**
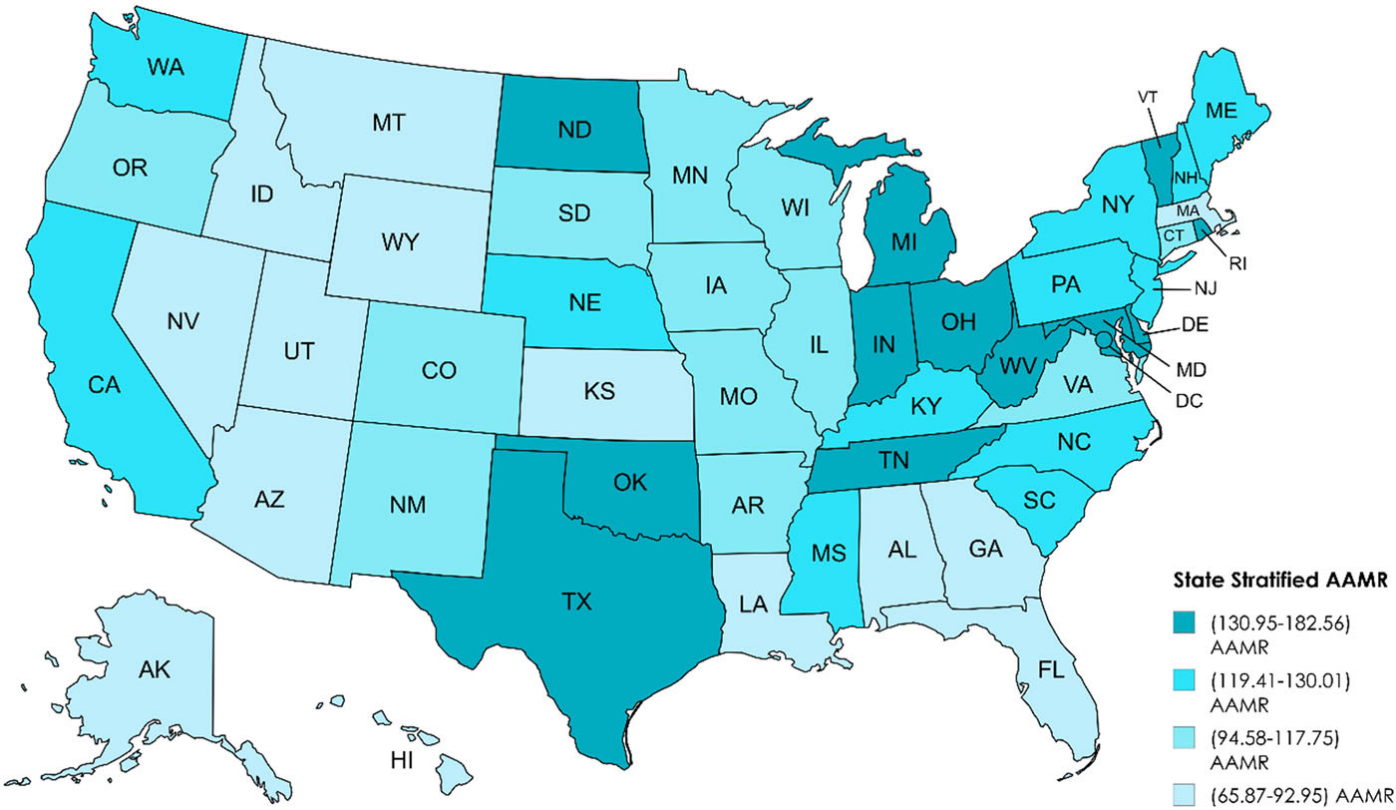
Dementia with coronary artery disease in older adults AAMR stratified by state per 100,000 population. Age‐adjusted mortality rate.

Variations in AAMRs were observed among different states, with AAMRs ranging from as low as 65.87 (95% CI: 63.79–67.95) in Nevada to 182.56 (95% CI: 180.04–185.07) in Oklahoma. States falling within the top 90th percentile included Rhode Island, Tennessee, Maryland, Ohio, and Vermont, which had approximately twice as high AAMRs compared to states in the lower 10th percentile, which included Utah, Arizona, Louisiana, Hawaii, and Georgia (Table S8; Figure 5).

### 3.5 Dementia With Coronary Artery Disease–Related AAMR Stratified by Subgroups

#### 3.5.1 Stratified by Vascular Dementia

From 1999 to 2023, overall AAMR for coronary artery disease–related vascular dementia among older adults increased with an AAPC of 8.49 (95% CI: 6.05–11.97; *p* value < 0.001). AAMR trend exhibited steepest decline between 1999 and 2001, followed by an upward trend from 2001 to 2005. Finally, AAMR trend rose between 2005 and 2023 (Table S9).

#### 3.5.2 Stratified by Unspecified Dementia

Overall, AAMR trend for coronary artery disease–related unspecified dementia among older adults exhibited a non‐significant plateaued trend throughout the study period with an AAPC of 0.05 (95% CI: −0.98 to 1.35; *p* value = 0.975). AAMR trend showed a rise between 1999 and 2001 and a subsequent decline by 2023 (Table S9).

#### 3.5.3 Stratified by Alzheimer's Disease

For coronary artery disease–related Alzheimer's disease, AAMR decreased with an AAPC of −2.29 (95% CI: −2.63 to −1.93; *p* value < 0.001). AAMR trend stabilized initially from 1999 to 2005 and declined between 2005 and 2014. Subsequently, AAMR trend increased between 2014 and 2021 and declined by 2023 (Table S9).

## 4 Discussion

The trend analysis from the CDC database from 1999 to 2023 revealed dynamic but important differences in CAD‐related mortality with dementia among older adults aged ≥65 years in the United States. The highest rate of mortality was present among older men as compared to older women. Moreover, the mortality rates were higher among NH Whites, followed by NH Blacks, Hispanics, NH American Indians or Alaska Natives, and NH Asians or Pacific Islanders. The states from the 90th percentile, including Rhode Island, Tennessee, Maryland, Ohio, and Vermont, showed overall double mortality rates as compared to the lower 10th percentile, including Utah, Arizona, Louisiana, Hawaii, and Georgia. Additionally, the individuals from the Midwest region showed higher mortality rates as compared to other regions. The subtype analysis confirmed that vascular dementia with CAD carries a higher mortality burden, likely reflecting overlapping vascular pathology and cerebrovascular mechanisms, whereas Alzheimer's and unspecified forms exhibited milder trends (Graphical Abstract).

Both CAD and dementia are among the major causes of death worldwide, particularly among older adults (Fadah et al. 2022; LoGiudice and Watson 2014). Dementia is a clinical syndrome characterized by a progressive loss of cognitive functions, including memory, thinking, judgment, and language, severe enough to interfere with normal social interactions and relationships (WHO 2019). The pathophysiology of CAD and dementia is intricately linked through vascular complications and neurodegenerative mechanisms. CAD is primarily driven by atheromatous plaque formation, which narrows the coronary arteries and leads to cardiac ischemia (Shahjehan et al. 2024). This, in turn, results in chronic hypoperfusion, disruption of the blood–brain barrier, activation of inflammatory cascades, and oxidative stress, ultimately contributing to amyloid‐beta accumulation and tau pathology—hallmarks of neurodegeneration (De La Torre 2012). Dementia, particularly Alzheimer's disease, is characterized by gradual neuronal loss, beta‐amyloid plaques, and neurofibrillary tangle accumulation (Nelson et al. 2008). Its progression is often exacerbated by CAD‐related comorbidities, including diabetes mellitus, hypertension, and dyslipidemia (Olesen et al. 2024). These bidirectional relationships between CAD and dementia highlight the importance of early identification and management of CAD‐related risk factors to help prevent chronic neurodegenerative diseases. The overall mortality rate due to CAD with dementia among older adults declined from 1999 to 2023, although the sharp rise between 1999 and 2001 may reflect, at least in part, an artifact of the initial ICD‐10 implementation, which temporarily influenced reporting of CAD and dementia, including Alzheimer's disease, a major cause of dementia (Anderson et al. 2001). The spike was short‐lived, as coding and reporting practices stabilized in subsequent years (Anderson et al. 2001). However, the subsequent decline in mortality trends may be attributable to better management of cardiovascular risk factors, increased awareness of dementia, and prompt treatment—factors that together have likely contributed to this reduction (Gorelick et al. 2011; Livingston et al. 2020).

Older men consistently showed higher mortality rates as compared to older women. This higher prevalence of deaths among older men may be presumably due to the higher prevalence of cardiovascular risk factors, including hypertension, diabetes mellitus, and atherosclerosis, which can directly contribute to the exacerbation of both cardiovascular and neurodegenerative pathology (Alyami et al. 2022; Man et al. 2020). In contrast, older women had lower mortality rates, potentially due to a lesser burden of comorbidities, more favorable vascular remodeling, and lower incidence of cardiovascular events (Alyami et al. 2022; Rodgers et al. 2019; Butters et al. 2021). Cardiac magnetic resonance imaging has shown that older women possess lower ventricular dimensions, lower ventricular mass, and preserved stroke volume and ejection fraction compared to older men, which saves older women from chronic hypoperfusion and subsequent development of neurodegenerative problems (Grassow et al. 2024). Both sexes have shown a spike in the mortality rates between 1999 and 2001 due to the same reason as explained above. In contrast to a previous study for Alzheimer's mortality trends, which found women to be at higher risk (Han et al. 2021), our study found older men to have higher mortality due to CAD with dementia, which might be due to more prevalence of CAD and cardiac‐related health conditions (Akhtar et al. 2025). Despite the lesser absolute mortality rates, older women showed relatively less stable APC, suggesting that access to health care, disease management, and reporting practices may disproportionately affect older women's outcomes (Amjad et al. 2018; Bosomworth and Khan 2023). Overall, these findings explain the complex interplay between cardiovascular risk factors, disease management, and neurodegeneration, which underscores the need for sex‐specific interventions and healthcare approaches to reduce the overall mortality, particularly among older women.

Significant differences were found among different ethnic and racial groups. NH Whites consistently showed higher mortality rates as compared to other races. This greater mortality burden among NH Whites may be partly attributed to their higher absolute prevalence of chronic health conditions such as diabetes, metabolic disorders, and cardiovascular disease, which are more common in older adults and may collectively elevate the risk of both neurodegenerative and cardiovascular mortality (Ali et al. 2025; Kramarow and Tejada‐Vera 2019). In addition, NH Whites form a larger proportion of the US population aged ≥65 years and particularly of the oldest‐old age groups, in whom mortality from CAD and dementia increases steeply (US Census Bureau 2024; Corrada et al. 2009; Santos et al. 2023). Consequently, their demographic structure and longer survival into advanced age may amplify AAMRs. Non‐Hispanic (NH) Black adults tend to develop chronic diseases earlier in midlife, resulting in higher disease burden in early older age. In contrast, NH Whites, who form the majority of the oldest population, survive longer with chronic conditions and gradually accumulate cardiometabolic and neurodegenerative risks (Corrada et al. 2009; Santos et al. 2023; Quiñones et al. 2019). This combination of longer survival and larger population share may contribute to higher AAMRs from CAD and dementia among older NH Whites. In contrast, Hispanics, NH Asians or Pacific Islanders, and NH American Indians or Alaska Natives showed relatively lower mortality rates, reflecting a lower burden of cardiovascular risk factors, healthier lifestyles, and protective genetic and community factors (Cockerham et al. 2016). These findings underscore the need for race‐specific multidimensional approaches to decrease the mortality rates, particularly among the vulnerable groups.

Marked interstate disparities exist in dementia with CAD‐related mortality among older adults. States in the top mortality percentile, such as Rhode Island, Tennessee, Maryland, Ohio, and Vermont, bear twice the burden of those in the lowest percentile, including Utah, Arizona, Louisiana, Hawaii, and Georgia. These state‐level disparities align with US Census region findings, in which the Midwest consistently exhibits the highest mortality profile, followed by the Northeast, South, and West. In contrast, prior studies have documented strong regional variation in dementia outcomes. Ailshire et al. (2022) reported subnational variation in dementia prevalence across US census regions, with South having the highest prevalence and the West the lowest. Similarly, Akushevich et al. (2021) found the highest Alzheimer's and related dementia mortality in the southeast, and Karway et al. (2025) reported the greatest fraction of cardiometabolic dementia cases in the similar regions. This divergence suggests that the patterns of disease incidence and mortality do not always coincide (Ailshire et al. 2022; Akushevich et al. 2021; Karway et al. 2025), and these regional mortality trends stabilized, with the most marked plateau observed in the Midwest. These regional trends of stabilization may reflect a combination of factors, including improvement in dementia detection and management, though notable regional differences remain (Hafiz et al. 2023).

Dementia with CAD‐related mortality is consistently higher in rural than urban areas, with the gap widening over two decades. This disparity may reflect a combination of higher vascular risk factor prevalence, delayed detection of cognitive impairment, and fewer emergency cardiac care centers in rural settings (Kapral et al. 2019; Wiese et al. 2014; Loccoh et al. 2022). Furthermore, the structural barriers, including specialist shortages, poorer health services, and limited rehabilitation infrastructure, further compound the rural risk (Liu et al. 2024; Douthit et al. 2015; Blank et al. 1996). In contrast, the relatively stable mortality profile among the urban population may reflect better access to emergency services (Carr et al. 2009).

The coexistence of dementia and CAD in older adults represents a compound public health challenge, with national mortality rates masking stark inequities by sex, race, geography, and rural–urban status. To address the intersecting burden of dementia and CAD, a multilayered and innovative approach is warranted. These strategies must include in‐house brain–heart clinics where neurology, geriatrics, and cardiology services are co‐located to reduce care fragmentation and enable real‐time multidisciplinary decision‐making (De La Torre 2014). AI‐driven early detections and wearables may detect concurrent cognitive and cardiac decline (Weil et al. 2022; Graham et al. 2019). In rural and underserved areas, using dementia–cardiac mobile phone applications with electrocardiography and cognitive screening tools can overcome access barriers and diagnostic delays (Eggink et al. 2021). Further strategies may include digital remote monitoring tools and lifestyle‐based vascular risk prevention (Christogianni 2023; Tariq and Barber 2017). Addressing the intertwined burden of dementia and CAD requires a paradigm shift—one that integrates vascular and cognitive care, leverages innovative technologies for early detection, and ensures equitable access across geographical and social divides. Without such decisive actions, the mortality gap will persist, and an aging population will bear the cost not only in years of life lost but also in years lived with disability.

### 4.1 Future Directions

Building on the practical strategies described previously, future research should focus on elucidating the biological mechanisms linking dementia and CAD, including the risk of vascular risk factors and potential biomarkers. Longitudinal studies are needed to evaluate the effectiveness of integrated brain–heart care models, as well as AI‐driven early detection tools and wearable technologies for simultaneous cognitive and cardiac monitoring (De La Torre 2014; Weil et al. 2022; Graham et al. 2019). Additional research should focus on reducing rural–urban and racial/ethnic disparities through mobile health interventions, remote monitoring applications, and lifestyle‐based vascular risk prevention tailored to the underserved population (Eggink et al. 2021; Christogianni 2023; Tariq and Barber 2017). Finally, studies assessing scalability, cost‐effectiveness, and implementation of these multidisciplinary and technology‐enabled interventions will be crucial to inform public health policy and improve equitable outcomes in older adults with dementia and CAD.

### 4.2 Limitations

Despite providing a comprehensive analysis of CAD‐related mortality in individuals with dementia, this study has several limitations. First, we relied on data from death certificates, which may lead to misclassification and underreporting of both CAD and dementia, potentially affecting accuracy. Moreover, detailed clinical information—such as medication use, lifestyle factors, and disease severity—was unavailable, yet is essential for a deeper understanding of mortality in this population. Although we examined sex‐, race‐, and geography‐based mortality trends, data on specific socioeconomic conditions, healthcare access, and other social determinants were not included, despite their potential contribution to observed differences. Additionally, data on urbanization were unavailable after 2020, limiting our ability to draw firm conclusions regarding mortality trends in rural versus urban populations.

## 5 Conclusion

From 1999 to 2023, the coexistence of dementia and CAD showed persistent mortality disparities across the United States. Overall, AAMRs stabilized, with older males experiencing an increase and older females a decline. Among racial and ethnic groups, NH White individuals had the highest mortality, followed by NH Black, Hispanic, NH American Indian/Alaska Native, and NH Asian/Pacific Islander. Regionally, the Midwest had the highest rates, whereas rural populations consistently exceeded urban ones. States such as Rhode Island, Tennessee, Maryland, Ohio, and Vermont showed roughly twice the mortality of lower burden states like Utah, Arizona, Louisiana, Hawaii, and Georgia. These findings highlight enduring inequalities and emphasize the need for targeted, evidence‐based interventions to improve health equity in older adults with dementia and CAD.

## Author Contributions

**Saifullah Khan**: conceptualization, data curation, project administration, formal analysis, writing – original draft. **Muhammad Hassan**: validation, visualization, writing – review and editing. **Muhammad Hussain**: writing – review and editing. **Wania Fatima**: literature search, writing – review and editing. **Javeria Nawaz**: methodology. **F. N. U. Pirih**: visualization and figures preparation. **Aiza Ahsan**: interpretation of results, manuscript editing. **Nisha Khalid**: writing – review and editing, visualization. **Mariam Qaisar**: visualization and figures preparation. **Sherif Eltawansy**: conceptual guidance, critical revision for intellectual content. **Muhammad Khalid Afridi**: review and editing. **Preet Memon**: data visualization. **Raheel Ahmed**: review of statistical analysis, supervision. **Hasibullah Aminpoor**: supervision, final approval of the version to be published, corresponding author responsibilities. All authors read and approved final version of manuscript.

## Funding

The authors have nothing to report.

## Ethics Statement

The authors have nothing to report.

## Conflicts of Interest

The authors declare no conflicts of interest.

## Supporting information




**Supplementary Table**: brb371125‐sup‐0001‐TableS1‐S11.docx

## Data Availability

The data supporting the findings of this study were obtained from the CDC WONDER online database (Centers for Disease Control and Prevention Wide‐ranging Online Data for Epidemiologic Research). The datasets used and analyzed during the current study are publicly available and can be accessed at [CDC WONDER] (https://wonder.cdc.gov).
