# Supplementary material for: Trends and Disparities in Dementia Among Older Adults With Coronary Artery Disease, 1999–2023: Insights From the CDC WONDER Database
Source: Brain Behav. 2025 Dec 17;15(12):e71125. doi: 10.1002/brb3.71125 (PMC12710092; doi:10.1002/brb3.71125)
Supplement: Supplementary file 1 — Supplementary Table: brb371125‐sup‐0001‐TableS1‐S11.docx [file BRB3-15-e71125-s001.docx]

**Supplemental Table 1, Dementia related mortalities in Older Adults with Coronary Artery Disease, Stratified by Sex and Race, in the United States, 1999 to 2023**

| **Supplemental Table 1, Dementia related mortalities in Older Adults with Coronary Artery Disease, Stratified by Sex and Race, in the United States, 1999 to 2023** | | | | | | | | | |
| --- | --- | --- | --- | --- | --- | --- | --- | --- | --- |
| **Deaths** | | | | | | | | | |
| **Year** | **Overall** | **Older Women** | **Older Men** | **NH American Indian or Alaska Native** | **NH Asian or Pacific Islander** | **NH Whites** | **NH Blacks** | **Hispanic** | **Population** |
| **1999** | 33499 | 22462 | 11037 | 54 | 240 | 29997 | 2343 | 788 | 34797841 |
| **2000** | 45563 | 30398 | 15165 | 94 | 389 | 40348 | 3388 | 1235 | 34991753 |
| **2001** | 47548 | 31204 | 16344 | 78 | 433 | 42065 | 3480 | 1367 | 35290291 |
| **2002** | 49240 | 32168 | 17072 | 106 | 469 | 43360 | 3719 | 1451 | 35522207 |
| **2003** | 50130 | 32407 | 17723 | 108 | 518 | 44152 | 3756 | 1499 | 35863529 |
| **2004** | 48752 | 31184 | 17568 | 99 | 528 | 42572 | 3794 | 1679 | 36203319 |
| **2005** | 50585 | 32146 | 18439 | 121 | 557 | 44262 | 3819 | 1763 | 36649798 |
| **2006** | 49813 | 31429 | 18384 | 140 | 646 | 43409 | 3701 | 1852 | 37164107 |
| **2007** | 50229 | 31261 | 18968 | 132 | 635 | 43715 | 3805 | 1896 | 37825711 |
| **2008** | 51057 | 31594 | 19463 | 124 | 647 | 44350 | 3812 | 2051 | 38777621 |
| **2009** | 49285 | 30020 | 19265 | 137 | 730 | 42598 | 3682 | 2068 | 39623175 |
| **2010** | 52069 | 31163 | 20906 | 145 | 843 | 44921 | 3745 | 2331 | 40267984 |
| **2011** | 52923 | 31724 | 21199 | 153 | 842 | 45576 | 3769 | 2507 | 41394141 |
| **2012** | 53091 | 31397 | 21694 | 181 | 911 | 45264 | 4022 | 2589 | 43145356 |
| **2013** | 52780 | 30908 | 21872 | 163 | 926 | 44835 | 3937 | 2800 | 44704074 |
| **2014** | 51047 | 29734 | 21313 | 155 | 927 | 43203 | 3785 | 2833 | 46243211 |
| **2015** | 50384 | 28955 | 21429 | 190 | 944 | 42602 | 3778 | 2726 | 47760852 |
| **2016** | 49138 | 27960 | 21178 | 178 | 981 | 41314 | 3768 | 2787 | 49244195 |
| **2017** | 50466 | 28245 | 22221 | 197 | 992 | 42435 | 3833 | 2885 | 50858679 |
| **2018** | 50358 | 27862 | 22496 | 180 | 1127 | 42159 | 3794 | 2996 | 52431193 |
| **2019** | 49949 | 27550 | 22399 | 188 | 1078 | 41516 | 3932 | 3142 | 54058263 |
| **2020** | 61769 | 33635 | 28134 | 215 | 1502 | 50155 | 5345 | 4421 | 55659365 |
| **2021** | 53551 | 28678 | 24873 | 183 | 1263 | 43931 | 4418 | 3509 | 55847953 |
| **2022** | 53709 | 28733 | 24976 | 204 | 1405 | 43948 | 4316 | 3587 | 57794852 |
| **2023** | 49349 | 26116 | 23233 | 227 | 1226 | 40507 | 3941 | 3187 | 59248361 |
| **Total** | **1256284** | **748933** | **507351** | **3752** | **20759** | **1073194** | **95682** | **59949** | **1101367831** |

**Supplemental Table 2, Dementia related Mortality, Stratified by Place of Death, in Older Adults with Coronary Artery Disease in the United States, 1999 to 2023**

| **Supplemental Table 2, Dementia related Mortality, Stratified by Place of Death, in Older Adults with Coronary Artery Disease in the United States, 1999 to 2023** | | | | |
| --- | --- | --- | --- | --- |
| **Deaths** | | | | |
| **Year** | **Medical Facility** | **Nursing Home/Long-term Care Facility** | **Hospice Facility** | **Decedent's Home** |
| **1999** | 8396 | 20894 | - | 3403 |
| **2000** | 11126 | 28475 | - | 4629 |
| **2001** | 11702 | 29212 | - | 5055 |
| **2002** | 11951 | 29965 | - | 5499 |
| **2003** | 11921 | 30142 | 61 | 5934 |
| **2004** | 11217 | 28834 | 112 | 6278 |
| **2005** | 11463 | 29884 | 534 | 6562 |
| **2006** | 10944 | 28968 | 737 | 7067 |
| **2007** | 11093 | 28612 | 1083 | 7207 |
| **2008** | 10980 | 28656 | 1254 | 7450 |
| **2009** | 9848 | 27012 | 1359 | 7948 |
| **2010** | 10302 | 28183 | 1645 | 9081 |
| **2011** | 10209 | 28404 | 2053 | 9357 |
| **2012** | 9828 | 28006 | 2289 | 9838 |
| **2013** | 9293 | 27599 | 2436 | 10187 |
| **2014** | 8775 | 26741 | 2490 | 10164 |
| **2015** | 8611 | 26313 | 2736 | 9974 |
| **2016** | 8163 | 24800 | 2775 | 10324 |
| **2017** | 8144 | 25724 | 3032 | 10458 |
| **2018** | 7873 | 25430 | 3010 | 10971 |
| **2019** | 7730 | 24505 | 3242 | 11308 |
| **2020** | 10007 | 28748 | 3837 | 15066 |
| **2021** | 8735 | 22094 | 3759 | 15218 |
| **2022** | 8822 | 22030 | 4052 | 14804 |
| **2023** | 7838 | 21144 | 3845 | 13296 |
| **Total** | **244971** | **670375** | **46341** | **227078** |

**Supplemental Table 3 Annual percent change (APC) of Dementia related Age-Adjusted Mortality Rates per 100,000, in Older Adults with Coronary Artery Disease in the United States, 1999 to 2023**

| **Supplemental Table 3 Annual percent change (APC) of Dementia related Age-Adjusted Mortality Rates per 100,000, in Older Adults with Coronary Artery Disease in the United States, 1999 to 2023** | | |
| --- | --- | --- |
| **Year Interval** | **APC (95% CI)** | **P-value** |
| **Overall** | | |
| 1999-2001 | 15.91* (2.45 to 25.67) | 0.019 |
| 2001-2023 | -1.59* (-2.07 to -1.24) | 0.002 |
| **Older Men** | | |
| 1999-2001 | 18.46* (1.98 to 33.82) | 0.019 |
| 2001-2023 | -0.84* (-1.43 to -0.42) | 0.005 |
| **Older Women** | | |
| 1999-2001 | 15.32* (3.55 to 22.56) | 0.009 |
| 2001-2023 | -2.15* (-2.57 to -1.83) | 0.0008 |
| **NH American Indian or Alaska Native** | | |
| 1999-2006 | 6.51* (2.21 to 18.96) | 0.008 |
| 2006-2023 | -2.24* (-3.52 to -1.26) | <0.001 |
| **NH Asian or Pacific Islander** | | |
| 1999-2001 | 23.00 (-2.12 to 62.25) | 0.227 |
| 2001-2023 | -1.66 (-10.90 to 0.46) | 0.062 |
| **NH White** | | |
| 1999-2001 | 15.01* (3.12 to 23.69) | 0.011 |
| 2001-2023 | -1.37* (-1.82 to -1.05) | 0.0008 |
| **NH Black** | | |
| 1999-2001 | 21.92* (0.37 to 38.85) | 0.043 |
| 2001-2023 | -2.00* (-2.91 to -1.47) | 0.007 |
| **Hispanic** | | |
| 1999-2001 | 21.09* (6.34 to 35.72) | 0.0008 |
| 2001-2013 | -0.32 (-1.28 to 0.82) | 0.544 |
| 2013-2017 | -6.98* (-11.64 to -4.17) | <0.001 |
| 2017-2020 | 10.00* (4.67 to 14.30) | <0.001 |
| 2020-2023 | -9.31* (-14.90 to -6.03) | <0.001 |
| **Rural areas** | | |
| 1999-2001 | 16.09* (9.03 to 21.38) | <0.001 |
| 2001-2010 | -0.30 (-1.00 to 1.18) | 0.499 |
| 2010-2018 | -2.29* (-5.50 to -1.63) | 0.0004 |
| 2018-2020 | 8.24* (2.45 to 12.38) | 0.001 |
| **Urban areas** | | |
| 1999-2001 | 17.86* (7.82 to 26.12) | <0.001 |
| 2001-2018 | -2.26* (-3.19 to -1.92) | 0.0004 |
| 2018-2020 | 6.46 (-1.04 to 10.68) | 0.123 |
| **Northeast region** | | |
| 1999-2001 | 17.16* (1.82 to 26.96) | 0.024 |
| 2001-2023 | -1.64* (-2.23 to -1.26) | 0.006 |
| **South region** | | |
| 1999-2001 | 11.41* (4.53 to 17.07) | <0.001 |
| 2001-2012 | -1.06 (-1.69 to 0.06) | 0.055 |
| 2012-2018 | -3.29* (-6.75 to -2.24) | 0.006 |
| 2018-2021 | 7.65* (4.06 to 10.44) | 0.007 |
| 2021-2023 | -7.41* (-11.46 to -2.71) | 0.008 |
| **Midwest region** | | |
| 1999-2001 | 17.18* (2.83 to 28.01) | 0.017 |
| 2001-2023 | -1.54* (-2.08 to -1.18) | 0.003 |
| **West region** | | |
| 1999-2001 | 25.06* (12.80 to 33.79) | <0.001 |
| 2001-2018 | -2.97* (-3.68 to -2.44) | 0.004 |
| 2018-2021 | 3.99 (-4.33 to 6.58) | 0.135 |
| 2021-2023 | -9.19* (-14.29 to -0.84) | 0.021 |
| APC = annual percent change; NH = non-Hispanic; * Indicates that the annual percentage change (APC) is significantly different from zero at α = 0.05. AAMR = age-adjusted mortality rate. The data for urbanization is only available till 2020 in the CDC Wonder Database. | | |

**Supplemental Table 4 Overall and Sex‐Stratified Dementia related Age-Adjusted Mortality Rates per 100,000, in Older Adults with Coronary Artery Disease in the United States, 1999 to 2023**

| **Supplemental Table 4 Overall and Sex‐Stratified Dementia related Age-Adjusted Mortality Rates per 100,000, in Older Adults with Coronary Artery Disease in the United States, 1999 to 2023** | | | |
| --- | --- | --- | --- |
| **Age-Adjusted Rate (95% CI)** | | | |
| **Year** | **Older Men** | **Older Women** | **Overall** |
| **1999** | 95.71 (93.89 - 97.52) | 97.8 (96.52 - 99.08) | 98.18 (97.13 - 99.23) |
| **2000** | 129.39 (127.3 - 131.48) | 130.7 (129.23 - 132.18) | 131.4 (130.19 - 132.6) |
| **2001** | 135.88 (133.76 - 137.99) | 132.43 (130.96 - 133.91) | 134.84 (133.63 - 136.06) |
| **2002** | 139.51 (137.39 - 141.63) | 135.33 (133.84 - 136.81) | 137.93 (136.71 - 139.14) |
| **2003** | 141.15 (139.04 - 143.25) | 134.52 (133.05 - 135.99) | 137.96 (136.76 - 139.17) |
| **2004** | 136.95 (134.9 - 139) | 128.42 (126.99 - 129.85) | 132.33 (131.16 - 133.51) |
| **2005** | 139.65 (137.61 - 141.68) | 129.71 (128.28 - 131.13) | 134.21 (133.04 - 135.38) |
| **2006** | 135.1 (133.13 - 137.06) | 124.29 (122.91 - 125.67) | 129 (127.87 - 130.14) |
| **2007** | 135.27 (133.34 - 137.21) | 121.12 (119.76 - 122.47) | 127.03 (125.92 - 128.15) |
| **2008** | 134.95 (133.05 - 136.85) | 120.61 (119.27 - 121.96) | 126.48 (125.38 - 127.58) |
| **2009** | 129.97 (128.13 - 131.81) | 112.4 (111.12 - 113.69) | 119.47 (118.41 - 120.53) |
| **2010** | 138.03 (136.15 - 139.9) | 114.64 (113.35 - 115.93) | 123.86 (122.79 - 124.93) |
| **2011** | 134.33 (132.52 - 136.15) | 113.74 (112.47 - 115.01) | 121.94 (120.89 - 122.98) |
| **2012** | 133.36 (131.58 - 135.14) | 110.35 (109.1 - 111.59) | 119.51 (118.49 - 120.54) |
| **2013** | 130.04 (128.31 - 131.76) | 106.83 (105.62 - 108.04) | 116.06 (115.06 - 117.06) |
| **2014** | 122.89 (121.23 - 124.54) | 101.05 (99.88 - 102.22) | 109.85 (108.89 - 110.81) |
| **2015** | 120.05 (118.43 - 121.66) | 96.57 (95.44 - 97.71) | 106.14 (105.2 - 107.07) |
| **2016** | 115.54 (113.98 - 117.1) | 92.05 (90.95 - 93.14) | 101.57 (100.66 - 102.47) |
| **2017** | 117.74 (116.18 - 119.29) | 91.64 (90.55 - 92.72) | 102.16 (101.26 - 103.06) |
| **2018** | 115.71 (114.19 - 117.23) | 89.04 (87.98 - 90.1) | 99.81 (98.93 - 100.69) |
| **2019** | 111.95 (110.48 - 113.43) | 87.04 (86 - 88.08) | 97.25 (96.39 - 98.11) |
| **2020** | 137.44 (135.83 - 139.05) | 105.26 (104.12 - 106.39) | 118.34 (117.4 - 119.28) |
| **2021** | 129.16 (127.54 - 130.78) | 97.24 (96.11 - 98.37) | 110.19 (109.25 - 111.12) |
| **2022** | 122.38 (120.85 - 123.91) | 89.17 (88.14 - 90.21) | 102.43 (101.56 - 103.3) |
| **2023** | 111.71 (110.26 - 113.16) | 83.87 (82.86 - 84.89) | 95.32 (94.47 - 96.16) |
| **Average** | **127.75 (125.96-129.54)** | **109.83 (108.58-111.09)** | **117.33 (116.30-118.36)** |

**Supplemental Table 5 Race‐Stratified Dementia related Age-Adjusted Mortality Rates per 100,000 in Older Adults with Coronary Artery Disease in the United States, 1999 to 2023**

| **Supplemental Table 5 Race‐Stratified Dementia related Age-Adjusted Mortality Rates per 100,000 in Older Adults with Coronary Artery Disease in the United States, 1999 to 2023** | | | | | |
| --- | --- | --- | --- | --- | --- |
| **Age-Adjusted Rate (95% CI)** | | | | | |
| **Year** | **NH American Indian or Alaska Native** | **NH Asian or Pacific Islander** | **NH Black** | **Hispanic** | **NH White** |
| **1999** | 53.1 (39.77 - 69.45) | 41.81 (36.43 - 47.19) | 91.41 (87.71 - 95.12) | 62.33 (57.94 - 66.71) | 101.27 (100.13 - 102.42) |
| **2000** | 83.53 (67.42 - 102.33) | 63.18 (56.82 - 69.55) | 130.42 (126.02 - 134.82) | 92.75 (87.54 - 97.96) | 134.42 (133.11 - 135.74) |
| **2001** | 67.4 (53.11 - 84.37) | 64.42 (58.28 - 70.56) | 133.3 (128.87 - 137.74) | 95.81 (90.68 - 100.94) | 138.12 (136.8 - 139.44) |
| **2002** | 92.4 (74.63 - 110.17) | 65.35 (59.37 - 71.33) | 142.18 (137.6 - 146.76) | 97.71 (92.63 - 102.79) | 140.84 (139.51 - 142.16) |
| **2003** | 90.45 (73.16 - 107.73) | 66.39 (60.61 - 72.18) | 142.63 (138.05 - 147.2) | 96.41 (91.48 - 101.34) | 141.27 (139.95 - 142.59) |
| **2004** | 81.91 (66.42 - 99.92) | 63.6 (58.13 - 69.08) | 142.19 (137.65 - 146.73) | 102.44 (97.49 - 107.39) | 134.77 (133.49 - 136.05) |
| **2005** | 96.37 (78.97 - 113.77) | 62.43 (57.21 - 67.65) | 139.55 (135.12 - 143.99) | 100.7 (95.95 - 105.44) | 137.41 (136.13 - 138.69) |
| **2006** | 108.36 (90.16 - 126.55) | 67.42 (62.19 - 72.66) | 132.68 (128.4 - 136.97) | 99.71 (95.13 - 104.29) | 131.88 (130.64 - 133.12) |
| **2007** | 99.52 (82.37 - 116.68) | 62.25 (57.38 - 67.11) | 133.2 (128.96 - 137.44) | 96.07 (91.72 - 100.42) | 130.21 (128.99 - 131.44) |
| **2008** | 87.3 (71.72 - 102.87) | 58.71 (54.17 - 63.26) | 129.73 (125.61 - 133.86) | 98.65 (94.36 - 102.94) | 130.02 (128.8 - 131.23) |
| **2009** | 94.18 (78.22 - 110.14) | 62.73 (58.17 - 67.3) | 121.21 (117.29 - 125.14) | 92.96 (88.94 - 96.99) | 122.74 (121.57 - 123.91) |
| **2010** | 97.08 (81.08 - 113.08) | 69.18 (64.5 - 73.86) | 120.34 (116.47 - 124.2) | 100.09 (96.01 - 104.17) | 127.4 (126.21 - 128.58) |
| **2011** | 96.52 (81.08 - 111.96) | 62.32 (58.1 - 66.54) | 117.19 (113.44 - 120.94) | 98.3 (94.44 - 102.15) | 126.04 (124.87 - 127.2) |
| **2012** | 106.01 (90.42 - 121.6) | 62.41 (58.35 - 66.47) | 119.66 (115.95 - 123.38) | 94.96 (91.29 - 98.63) | 123.16 (122.02 - 124.31) |
| **2013** | 90.5 (76.5 - 104.49) | 58.07 (54.32 - 61.82) | 113.49 (109.93 - 117.05) | 96.18 (92.61 - 99.75) | 119.97 (118.85 - 121.1) |
| **2014** | 79.46 (66.82 - 92.11) | 53.86 (50.38 - 57.33) | 105.03 (101.67 - 108.39) | 90.4 (87.07 - 93.74) | 114.13 (113.05 - 115.22) |
| **2015** | 91.19 (78.09 - 104.3) | 50.75 (47.5 - 53.99) | 100.92 (97.69 - 104.16) | 81.53 (78.46 - 84.6) | 111.9 (109.93 - 112.06) |
| **2016** | 80.63 (68.65 - 92.6) | 49.24 (46.15 - 52.33) | 97.75 (94.61 - 100.9) | 78.79 (75.86 - 81.73) | 106.36 (105.33 - 107.4) |
| **2017** | 85.5 (73.48 - 97.52) | 46.3 (43.41 - 49.19) | 95.92 (92.87 - 98.98) | 76.99 (74.17 - 79.81) | 107.86 (106.82 - 108.89) |
| **2018** | 71.6 (61.03 - 82.16) | 49.74 (46.83 - 52.66) | 91.72 (88.78 - 94.65) | 76.09 (73.35 - 78.83) | 105.55 (104.54 - 106.57) |
| **2019** | 72.51 (62.07 - 82.95) | 45.15 (42.45 - 47.86) | 91.96 (89.06 - 94.85) | 77.12 (74.41 - 79.83) | 102.69 (101.7 - 103.68) |
| **2020** | 78.01 (67.52 - 88.51) | 58.85 (55.86 - 61.84) | 121.65 (118.36 - 124.93) | 103.45 (100.39 - 106.51) | 123.12 (121.92 - 124.08) |
| **2021** | 72.28 (61.68 - 82.87) | 52.13 (49.24 - 55.01) | 106.16 (102.99 - 109.32) | 84.33 (81.53 - 87.14) | 117.79 (116.68 - 118.89) |
| **2022** | 71.53 (61.64 - 81.41) | 52.2 (49.46 - 54.94) | 98.73 (95.76 - 101.7) | 80.81 (78.15 - 83.46) | 109.12 (108.1 - 110.15) |
| **2023** | 74.57 (64.79 - 84.34) | 44.29 (41.81 - 46.78) | 89.03 (86.22 - 91.84) | 70.94 (68.46 - 73.41) | 102.76 (101.75 - 103.76) |
| **Average** | **84.88 (70.83-99.36)** | **57.31 (53.08-61.54)** | **116.32 (112.60-120.04)** | **89.82 (86.00-93.64)** | **121.59 (120.44-122.75)** |
| NH = Non-Hispanic  **Supplemental Table 6, Dementia related Age-Adjusted Mortality Rates per 100,000, Stratified by Census Region, in Older Adults with Coronary Artery Disease in the United States, 1999 to 2023**   \| **Supplemental Table 6, Dementia related Age-Adjusted Mortality Rates per 100,000, Stratified by Census Region, in Older Adults with Coronary Artery Disease in the United States, 1999 to 2023** \| \| \| \| \| \| --- \| --- \| --- \| --- \| --- \| \|  \| **Census Region: Northeast** \| **Census Region: Midwest** \| **Census Region: South** \| **Census Region: West** \| \| **Year** \| **Age-Adjusted Rate (95% CI)** \| **Age-Adjusted Rate (95% CI)** \| **Age-Adjusted Rate (95% CI)** \| **Age-Adjusted Rate (95% CI)** \| \| **1999** \| 97.14 (94.91 - 99.38) \| 97.9 (95.79 - 100.02) \| 105.1 (103.24 - 106.96) \| 87.11 (84.85 - 89.37) \| \| **2000** \| 130.5 (127.93 - 133.08) \| 140 (137.49 - 142.51) \| 125.19 (123.18 - 127.21) \| 132.29 (129.53 - 135.04) \| \| **2001** \| 135.5 (132.91 - 138.1) \| 140.72 (138.21 - 143.22) \| 128.7 (126.67 - 130.72) \| 137.26 (134.49 - 140.03) \| \| **2002** \| 139.96 (137.33 - 142.58) \| 141.62 (139.12 - 144.11) \| 132.89 (130.84 - 134.94) \| 139.31 (136.55 - 142.06) \| \| **2003** \| 133.77 (131.23 - 136.32) \| 142.53 (140.04 - 145.01) \| 135.25 (133.2 - 137.31) \| 141.17 (138.43 - 143.91) \| \| **2004** \| 133.12 (130.6 - 135.65) \| 136.39 (133.97 - 138.82) \| 127.61 (125.63 - 129.6) \| 134.23 (131.58 - 136.87) \| \| **2005** \| 132.86 (130.36 - 135.36) \| 140.75 (138.31 - 143.19) \| 130.48 (128.5 - 132.46) \| 133.6 (131.01 - 136.2) \| \| **2006** \| 126.75 (124.33 - 129.17) \| 135.7 (133.33 - 138.07) \| 124.66 (122.75 - 126.57) \| 130 (127.48 - 132.52) \| \| **2007** \| 125.39 (123.01 - 127.78) \| 136.01 (133.66 - 138.37) \| 124.08 (122.2 - 125.96) \| 122.46 (120.05 - 124.88) \| \| **2008** \| 122.38 (120.04 - 124.72) \| 135.93 (133.59 - 138.27) \| 124.29 (122.43 - 126.15) \| 122.86 (120.47 - 125.24) \| \| **2009** \| 117.9 (115.62 - 120.18) \| 124.73 (122.5 - 126.96) \| 120.69 (118.88 - 122.5) \| 112.6 (110.35 - 114.85) \| \| **2010** \| 127.83 (125.47 - 130.19) \| 127.06 (124.82 - 129.29) \| 124.6 (122.78 - 126.43) \| 114.37 (112.12 - 116.61) \| \| **2011** \| 125.52 (123.2 - 127.84) \| 127.38 (125.16 - 129.6) \| 120.55 (118.79 - 122.31) \| 113.93 (111.73 - 116.12) \| \| **2012** \| 123.19 (120.91 - 125.48) \| 124.72 (122.54 - 126.9) \| 119.27 (117.55 - 121) \| 109.78 (107.65 - 111.9) \| \| **2013** \| 118.76 (116.53 - 120.99) \| 120.92 (118.79 - 123.05) \| 116.94 (115.25 - 118.62) \| 105.98 (103.92 - 108.04) \| \| **2014** \| 113.19 (111.03 - 115.36) \| 117.36 (115.27 - 119.44) \| 109.94 (108.33 - 111.56) \| 97.96 (96.01 - 99.91) \| \| **2015** \| 110.52 (108.39 - 112.64) \| 112.91 (110.88 - 114.95) \| 105.55 (103.99 - 107.11) \| 94.93 (93.04 - 96.82) \| \| **2016** \| 104.31 (102.25 - 106.37) \| 107.57 (105.6 - 109.55) \| 100.74 (99.24 - 102.24) \| 93.54 (91.69 - 95.4) \| \| **2017** \| 105.58 (103.54 - 107.62) \| 107.85 (105.89 - 109.81) \| 101.91 (100.42 - 103.4) \| 92.82 (90.99 - 94.65) \| \| **2018** \| 105.22 (103.19 - 107.25) \| 105.79 (103.87 - 107.7) \| 100.06 (98.6 - 101.51) \| 87.74 (85.99 - 89.5) \| \| **2019** \| 98.04 (96.09 - 99.99) \| 104.38 (102.48 - 106.27) \| 98.63 (97.2 - 100.06) \| 86.63 (84.9 - 88.35) \| \| **2020** \| 123.61 (121.44 - 125.79) \| 127.89 (125.79 - 129.98) \| 121.44 (119.88 - 123) \| 98.55 (96.74 - 100.37) \| \| **2021** \| 102.65 (100.61 - 104.68) \| 113.97 (111.91 - 116.02) \| 117.78 (116.18 - 119.37) \| 99.91 (98.01 - 101.81) \| \| **2022** \| 95.27 (93.37 - 97.17) \| 106.03 (104.12 - 107.94) \| 110.93 (109.44 - 112.41) \| 90.61 (88.88 - 92.34) \| \| **2023** \| 87.08 (85.25 - 88.92) \| 99.43 (97.56 - 101.29) \| 104.77 (103.32 - 106.22) \| 82.32 (80.66 - 83.98) \| \| **Average** \| **117.44 (115.18-119.70)** \| **123.02 (120.83- 125.21)** \| **117.28 (115.54-119.08)** \| **110.48 (108.28-112.67)** \| | | | | | |

**Supplemental Table 7, Dementia related Age-Adjusted Mortality Rates per 100,000, Stratified by Urban-Rural Classification, in Older Adults with Coronary Artery Disease in the United States, 1999 to 2020**

| **Supplemental Table 7, Dementia related Age-Adjusted Mortality Rates per 100,000, Stratified by Urban-Rural Classification, in Older Adults with Coronary Artery Disease in the United States, 1999 to 2020** | | |
| --- | --- | --- |
| **Age-Adjusted Rate (95% CI)** | | |
| **Year** | **Urban** | **Rural** |
| **1999** | 97.97 (96.8 - 99.15) | 99.13 (96.76 - 101.51) |
| **2000** | 131.56 (130.21 - 132.9) | 130.64 (127.92 - 133.35) |
| **2001** | 135.35 (133.99 - 136.7) | 132.98 (130.25 - 135.71) |
| **2002** | 137.36 (136.01 - 138.71) | 140.26 (137.46 - 143.06) |
| **2003** | 136.68 (135.34 - 138.01) | 143.79 (140.96 - 146.61) |
| **2004** | 131.58 (130.28 - 132.88) | 135.69 (132.95 - 138.43) |
| **2005** | 132.51 (131.22 - 133.8) | 141.77 (138.99 - 144.56) |
| **2006** | 127.22 (125.98 - 128.47) | 136.74 (134.02 - 139.45) |
| **2007** | 124.48 (123.26 - 125.7) | 138.39 (135.68 - 141.1) |
| **2008** | 123.26 (122.06 - 124.46) | 140.91 (138.19 - 143.64) |
| **2009** | 116.15 (115.1 - 117.31) | 134.64 (131.99 - 137.29) |
| **2010** | 121.17 (120.1 - 122.33) | 136.26 (133.61 - 138.91) |
| **2011** | 119.5 (118.36 - 120.64) | 133.04 (130.44 - 135.63) |
| **2012** | 116.8 (115.69 - 117.92) | 132.01 (129.42 - 134.57) |
| **2013** | 113.71 (112.62 - 114.8) | 127.11 (124.62 - 129.61) |
| **2014** | 106.74 (105.7 - 107.78) | 124.38 (121.92 - 126.83) |
| **2015** | 102.81 (101.8 - 103.82) | 121.85 (119.43 - 124.26) |
| **2016** | 98.24 (97.26 - 99.21) | 117.65 (115.29 - 120.01) |
| **2017** | 99.01 (98.04 - 99.98) | 117.54 (115.21 - 119.87) |
| **2018** | 96.44 (95.5 - 97.39) | 116.34 (114.04 - 118.65) |
| **2019** | 93.21 (92.29 - 94.13) | 117.04 (114.75 - 119.33) |
| **2020** | 114.82 (113.81 - 115.83) | 135.9 (133.44 - 138.36) |
| **Average** | **117.12 (115.96-118.27)** | **129.73 (127.15-132.31)** |
| The data for urbanization is only available till 2020 in the CDC Wonder Database. | | |
|  |  |  |

**Supplemental Table 8, Dementia related Age-Adjusted Mortality Rates per 100,000, Stratified by States, in Older Adults with Coronary Artery Disease in the United States, 1999 to 2023**

| **Supplemental Table 8, Dementia related Age-Adjusted Mortality Rates per 100,000, Stratified by States, in Older Adults with Coronary Artery Disease in the United States, 1999 to 2023** | |
| --- | --- |
| **State** | **Age-Adjusted Rate (95% CI)** |
| Alabama | 102.29 (100.6 - 103.98) |
| Alaska | 87.44 (81.38 - 93.51) |
| Arizona | 78.5 (77.24 - 79.77) |
| Arkansas | 104.3 (102.2 - 106.4) |
| California | 122.48 (121.79 - 123.16) |
| Colorado | 107.96 (106.09 - 109.82) |
| Connecticut | 113.83 (112 - 115.67) |
| Delaware | 140.22 (135.79 - 144.64) |
| District of Columbia | 125.84 (120.47 - 131.21) |
| Florida | 92.95 (92.27 - 93.63) |
| Georgia | 83.14 (81.9 - 84.38) |
| Hawaii | 78.11 (75.62 - 80.6) |
| Idaho | 92.36 (89.47 - 95.26) |
| Illinois | 100.4 (99.4 - 101.41) |
| Indiana | 131.16 (129.54 - 132.78) |
| Iowa | 116.6 (114.64 - 118.55) |
| Kansas | 91.05 (89.13 - 92.97) |
| Kentucky | 130.01 (127.99 - 132.03) |
| Louisiana | 73.22 (71.69 - 74.74) |
| Maine | 120.98 (117.89 - 124.08) |
| Maryland | 159.67 (157.72 - 161.62) |
| Massachusetts | 88.47 (87.25 - 89.69) |
| Michigan | 137.03 (135.74 - 138.33) |
| Minnesota | 117.75 (116.12 - 119.38) |
| Mississippi | 119.87 (117.49 - 122.25) |
| Missouri | 114.03 (112.52 - 115.53) |
| Montana | 80.59 (77.54 - 83.65) |
| Nebraska | 121.26 (118.52 - 124) |
| Nevada | 65.87 (63.79 - 67.95) |
| New Hampshire | 119.41 (116.07 - 122.75) |
| New Jersey | 122.67 (121.39 - 123.95) |
| New Mexico | 117.69 (114.87 - 120.5) |
| New York | 120.98 (120.13 - 121.83) |
| North Carolina | 126.97 (125.6 - 128.34) |
| North Dakota | 131.47 (127.07 - 135.86) |
| Ohio | 159.94 (158.66 - 161.21) |
| Oklahoma | 182.56 (180.04 - 185.07) |
| Oregon | 110.35 (108.5 - 112.2) |
| Pennsylvania | 125.44 (124.45 - 126.43) |
| Rhode Island | 182.02 (177.87 - 186.17) |
| South Carolina | 123.39 (121.46 - 125.32) |
| South Dakota | 116.49 (112.64 - 120.35) |
| Tennessee | 153.25 (151.43 - 155.07) |
| Texas | 130.95 (130 - 131.91) |
| Utah | 67.27 (65.07 - 69.46) |
| Vermont | 175.86 (170.17 - 181.55) |
| Virginia | 94.58 (93.27 - 95.89) |
| Washington | 128.03 (126.41 - 129.64) |
| West Virginia | 175.17 (171.92 - 178.41) |
| Wisconsin | 113.98 (112.46 - 115.49) |
| Wyoming | 87.34 (82.63 - 92.05) |

**Supplemental Table 9 Subgroup‐Stratified Dementia related Age-Adjusted Mortality Rates per 100,000, in Older Adults with Coronary Artery Disease in the United States, 1999 to 2023**

| **Supplemental Table 9 Subgroup‐Stratified Dementia related Age-Adjusted Mortality Ratesper 100,000, in Older Adults with Coronary Artery Disease in the United States, 1999 to 2023** | | | |
| --- | --- | --- | --- |
| **Age-Adjusted Rate (95% CI)** | | | |
| **Year** | **Vascular Dementia** | **Unspecified Dementia** | **Alzheimer's Disease** |
| **1999** | 1.91 (1.77 - 2.06) | 52.84 (52.07 - 53.61) | 44.93 (44.22 - 45.65) |
| **2000** | 0.65 (0.56 - 0.73) | 87.81 (86.82 - 88.79) | 45.24 (44.53 - 45.95) |
| **2001** | 0.50 (0.42 - 0.58) | 90.79 (89.79 - 91.78) | 45.88 (45.17 - 46.59) |
| **2002** | 0.11 (0.08 - 0.15) | 91.98 (90.98 - 92.97) | 48.13 (47.41 - 48.85) |
| **2003** | - | 92.85 (91.86 - 93.84) | 47.00 (46.30 - 47.71) |
| **2004** | 0.08 (0.05 - 0.12) | 87.67 (86.71 - 88.63) | 46.34 (45.64 - 47.03) |
| **2005** | 7.19 (6.92 - 7.46) | 82.10 (81.18 - 83.01) | 46.60 (45.91 - 47.29) |
| **2006** | 8.78 (8.49 - 9.08) | 78.73 (77.85 - 79.62) | 44.05 (43.39 - 44.71) |
| **2007** | 7.01 (6.75 - 7.27) | 79.14 (78.26 - 80.02) | 42.65 (42.00 - 43.29) |
| **2008** | 7.70 (7.43 - 7.97) | 79.90 (79.03 - 80.77) | 40.94 (40.32 - 41.57) |
| **2009** | 7.37 (7.11 - 7.64) | 75.95 (75.11 - 76.80) | 37.87 (37.27 - 38.47) |
| **2010** | 8.01 (7.74 - 8.28) | 80.30 (79.44 - 81.16) | 37.53 (36.94 - 38.12) |
| **2011** | 7.79 (7.53 - 8.05) | 80.72 (79.87 - 81.57) | 35.08 (34.51 - 35.64) |
| **2012** | 7.92 (7.66 - 8.19) | 81.09 (80.25 - 81.94) | 32.20 (31.67 - 32.73) |
| **2013** | 8.30 (8.03 - 8.56) | 78.50 (77.68 - 79.32) | 30.76 (30.25 - 31.28) |
| **2014** | 8.56 (8.29 - 8.83) | 73.88 (73.09 - 74.66) | 28.91 (28.42 - 29.41) |
| **2015** | 8.57 (8.30 - 8.83) | 69.27 (68.52 - 70.03) | 29.91 (29.41 - 30.40) |
| **2016** | 9.18 (8.91 - 9.45) | 63.71 (63.00 - 64.43) | 30.48 (29.99 - 30.98) |
| **2017** | 10.04 (9.76 - 10.32) | 63.61 (62.90 - 64.32) | 30.30 (29.81 - 30.79) |
| **2018** | 10.61 (10.32 - 10.90) | 61.55 (60.86 - 62.24) | 29.61 (29.13 - 30.08) |
| **2019** | 10.97 (10.68 - 11.25) | 59.67 (59.00 - 60.34) | 28.69 (28.22 - 29.15) |
| **2020** | 13.27 (12.96 - 13.59) | 73.81 (73.08 - 74.55) | 33.95 (33.45 - 34.45) |
| **2021** | 13.10 (12.78 - 13.42) | 68.45 (67.71 - 69.18) | 31.42 (30.92 - 31.92) |
| **2022** | 13.04 (12.73 - 13.35) | 64.02 (63.34 - 64.71) | 28.30 (27.84 - 28.75) |
| **2023** | 12.52 (12.22 - 12.83) | 59.47 (58.81 - 60.14) | 26.19 (25.75 - 26.63) |

| ***Demographics*** | ***Deaths*** | ***Population*** | ***Mean Age-Adjusted Mortality Rate per 100,000 (95% CI)*** | ***AAPC (95% CI)*** |
| --- | --- | --- | --- | --- |
| *Overall* | 1,256,284 | 1101367831 | 117.33 (116.30 to 118.36) | -0.23 (-0.99 to 0.40) |
| ***Sex*** |  |  |  |  |
| Older Male | 507,351 | 478768966 | 127.75 (125.96 to 129.54) | 0.63 (-0.23 to 1.63) |
| Older Female | 748,933 | 622598865 | 109.83 (108.58 to 111.09) | -0.80* (-1.46 to -0.32) |
| ***Race*** |  |  |  |  |
| NH American Indian or Alaska Native | 3,752 | 5784353 | 84.88 (70.83 to 99.36) | 0.23 (-0.59 to 1.90) |
| NH Asian or Pacific Islander | 20,759 | 43981649 | 57.31 (53.08 to 61.54) | 0.18 (-2.06 to 2.53) |
| NH White | 1,073,194 | 871952155 | 121.59 (120.44 to 122.75) | -0.10 (-0.75 to 0.47) |
| NH Black | 95,682 | 97083093 | 116.32 (112.60 to 120.04) | -0.20 (-1.45 to 0.85) |
| Hispanic | 59,949 | 81040188 | 89.82 (86.00 to 93.64) | 0.19 (-0.57 to 1.13) |
| ***Census Region*** |  |  |  |  |
| South | 442,561 | 409059178 | 117.28 (115.54 to 119.08) | -0.14 (-0.56 to 0.28) |
| West | 246,600 | 237052954 | 110.48 (108.28 to 112.67) | -0.58 (-1.13 to 0.00) |
| Midwest | 304,965 | 243862323 | 123.02 (120.83 to 125.21) | -0.10 (-0.91 to 0.59) |
| Northeast | 262,158 | 211393376 | 117.44 (115.18 to 119.70) | -0.19 (-1.10 to 0.44) |
| ***Urbanization*** |  |  |  |  |
| Rural | 215,401 | 167978547 | 129.73 (127.15 to 132.31) | 1.16* (0.72 to 1.59) |
| Urban | 884,274 | 760496648 | 117.12 (115.96 to 118.27) | 0.30 (-0.32 to 0.92) |

* Indicates that the AAPC is statistically significant i.e. p value <0.05

**Supplemental Table 10. AAMR and AAPC for Dementia-related deaths among U.S. adults aged ≥ 65 years with CAD, 1999 to 2023.**

**Supplemental Table 11: STROBE Checklist**

STROBE Statement—checklist of items that should be included in reports of observational studies

|  | Item No. | Recommendation | Page  No. | Relevant text from manuscript |
| --- | --- | --- | --- | --- |
| **Title and abstract** | 1 | (*a*) Indicate the study’s design with a commonly used term in the title or the abstract |  | “A population-based cross-sectional trend analysis using CDC WONDER Multiple Cause-of-Death data (1999–2023).” (Abstract – Methods) |
|  |  | (*b*) Provide in the abstract an informative and balanced summary of what was done and what was found |  | “Abstract includes structured sections (Introduction, Methods, Results, Conclusion) summarizing rationale, dataset, analytic approach, key findings, and implications.” |
| Introduction | | | |  |
| Background/rationale | 2 | Explain the scientific background and rationale for the investigation being reported | “Introduction – Explains the underrepresentation of dementia populations in cardiovascular research and the need to study combined dementia–CAD mortality trends.” |  |
| Objectives | 3 | State specific objectives, including any prespecified hypotheses | “To examine 25-year nationwide trends in dementia and CAD-related mortality among elderly individuals in the United States.” (Introduction – last paragraph) |  |
| Methods | | | |  |
| Study design | 4 | Present key elements of study design early in the paper | “A descriptive cross-sectional trend analysis of national mortality data was conducted using CDC WONDER.” (Methods – first paragraph) |  |
| Setting | 5 | Describe the setting, locations, and relevant dates, including periods of recruitment, exposure, follow-up, and data collection | “U.S. population-based mortality data from 1999–2023 were obtained from CDC WONDER Multiple Cause-of-Death database.” (Methods – Data source) |  |
| Participants | 6 | (*a*) *Cohort study*—Give the eligibility criteria, and the sources and methods of selection of participants. Describe methods of follow-up  *Case-control study*—Give the eligibility criteria, and the sources and methods of case ascertainment and control selection. Give the rationale for the choice of cases and controls  *Cross-sectional study*—Give the eligibility criteria, and the sources and methods of selection of participants | “Included all decedents aged ≥65 years with ICD-10 codes I20–I25 (CAD) and F01, F03, or G30 (dementia) listed anywhere on the death certificate.” (Methods – Data abstraction) |  |
|  |  | (*b*) *Cohort study*—For matched studies, give matching criteria and number of exposed and unexposed  *Case-control study*—For matched studies, give matching criteria and the number of controls per case | “Not applicable – analysis based on de-identified national mortality dataset (aggregate counts).” |  |
| Variables | 7 | Clearly define all outcomes, exposures, predictors, potential confounders, and effect modifiers. Give diagnostic criteria, if applicable | “Primary variable: Age-adjusted mortality rate (AAMR) per 100,000 population for deaths with coexisting dementia and CAD. Variables stratified by sex, race/ethnicity, region, urban/rural status, and place of death.” |  |
| Data sources/ measurement | 8* | For each variable of interest, give sources of data and details of methods of assessment (measurement). Describe comparability of assessment methods if there is more than one group | *“Data derived from CDC WONDER Multiple Cause-of-Death database; AAMRs standardized to the 2000 U.S. population; dementia and CAD identified via ICD-10 codes.”* |  |
| Bias | 9 | Describe any efforts to address potential sources of bias | “Potential misclassification bias minimized by using standardized ICD-10 coding and consistent inclusion criteria across years.” |  |
| Study size | 10 | Explain how the study size was arrived at | “All eligible national death records (N = 1,256,284) between 1999 and 2023 were included; no sampling applied.” |  |

Continued on next page

| Quantitative variables | 11 | Explain how quantitative variables were handled in the analyses. If applicable, describe which groupings were chosen and why | “AAMRs calculated per 100,000; subgroup-specific average AAMRs obtained using Excel AVERAGE function; trends assessed via Joinpoint regression for APC and AAPC.” |  |
| --- | --- | --- | --- | --- |
| Statistical methods | 12 | (*a*) Describe all statistical methods, including those used to control for confounding | “Joinpoint regression used to calculate APC, AAPC, and 95% CIs; weighted BIC for model selection; empirical quantile method for CIs; significance threshold p < 0.05.” |  |
|  |  | (*b*) Describe any methods used to examine subgroups and interactions | “Subgroup analyses performed by sex, race/ethnicity, census region, place of death, and urban/rural status. No missing data as analysis used complete CDC WONDER records.” |  |
|  |  | (*c*) Explain how missing data were addressed | - |  |
|  |  | (*d*) *Cohort study*—If applicable, explain how loss to follow-up was addressed  *Case-control study*—If applicable, explain how matching of cases and controls was addressed  *Cross-sectional study*—If applicable, describe analytical methods taking account of sampling strategy | - |  |
|  |  | (*e*) Describe any sensitivity analyses | - |  |
| Results | | | | |
| Participants | 13* | (a) Report numbers of individuals at each stage of study—eg numbers potentially eligible, examined for eligibility, confirmed eligible, included in the study, completing follow-up, and analysed | “Not applicable – population-level aggregate data.” |  |
|  |  | (b) Give reasons for non-participation at each stage |  |  |
|  |  | (c) Consider use of a flow diagram |  |  |
| Descriptive data | 14* | (a) Give characteristics of study participants (eg demographic, clinical, social) and information on exposures and potential confounders | “Total of 1,256,284 deaths aged ≥65 years. Over half occurred in long-term care institutions (53.4%), followed by medical settings (19.5%), home (18.1%), other (5.2%), and hospice (3.7%).” (Abstract – Results) |  |
|  |  | (b) Indicate number of participants with missing data for each variable of interest | None |  |
|  |  | (c) *Cohort study*—Summarise follow-up time (eg, average and total amount) |  |  |
| Outcome data | 15* | *Cohort study*—Report numbers of outcome events or summary measures over time | *“1,256,284 dementia-with-CAD deaths (1999–2023).”* |  |
|  |  | *Case-control study—*Report numbers in each exposure category, or summary measures of exposure |  |  |
|  |  | *Cross-sectional study—*Report numbers of outcome events or summary measures |  |  |
| Main results | 16 | (*a*) Give unadjusted estimates and, if applicable, confounder-adjusted estimates and their precision (eg, 95% confidence interval). Make clear which confounders were adjusted for and why they were included | “AAMR trends and AAPC estimates with 95% CIs and p-values reported by demographic subgroups.” |  |
|  |  | (*b*) Report category boundaries when continuous variables were categorized | “Age category defined as ≥65 years; race/ethnicity categories per CDC WONDER definitions (NH White, NH Black, Hispanic, NH AI/AN, NH Asian or Pacific Islander).” |  |
|  |  | (*c*) If relevant, consider translating estimates of relative risk into absolute risk for a meaningful time period |  |  |

Continued on next page

| Other analyses | 17 | Report other analyses done—eg analyses of subgroups and interactions, and sensitivity analyses | “Subgroup analyses by dementia subtype (Alzheimer’s, vascular, unspecified) and geographic stratifications.” |  |
| --- | --- | --- | --- | --- |
| Discussion | | | | |
| Key results | 18 | Summarise key results with reference to study objectives | “Overall AAMR trend remained stable; male rates higher than female; NH Whites highest among races; rural areas showed significant upward trend.” (Abstract – Results) |  |
| Limitations | 19 | Discuss limitations of the study, taking into account sources of potential bias or imprecision. Discuss both direction and magnitude of any potential bias | “Acknowledges limitations of death certificate data, potential diagnostic misclassification, and ecological nature of CDC WONDER data.” (Discussion – Limitations paragraph) |  |
| Interpretation | 20 | Give a cautious overall interpretation of results considering objectives, limitations, multiplicity of analyses, results from similar studies, and other relevant evidence | “Findings suggest persistent mortality burden from dementia with CAD, particularly in males, rural regions, and long-term care settings; underscores need for inclusive research.” (Conclusion) |  |
| Generalisability | 21 | Discuss the generalisability (external validity) of the study results | “Results generalizable to the U.S. elderly population due to complete nationwide data coverage.” |  |
| Other information | |  | | |
| Funding | 22 | Give the source of funding and the role of the funders for the present study and, if applicable, for the original study on which the present article is based | “No external funding was received for this study.” (Declarations/Funding section) |  |

**Note:** An Explanation and Elaboration article discusses each checklist item and gives methodological background and published examples of transparent reporting. The STROBE checklist is best used in conjunction with this article (freely available on the Web sites of PLoS Medicine at http://www.plosmedicine.org/, Annals of Internal Medicine at http://www.annals.org/, and Epidemiology at http://www.epidem.com/). Information on the STROBE Initiative is available at www.strobe-statement.org.
